# Comparative transcriptomics and network analysis define gene coexpression modules that control maize aleurone development and auxin signaling

**DOI:** 10.1002/tpg2.20126

**Published:** 2021-07-29

**Authors:** Hao Wu, Philip W. Becraft

**Affiliations:** ^1^ Dep. of Genetics Development & Cell Biology, IA State Univ. Ames IA 50011 USA; ^2^ Agronomy Dep. IA State Univ. Ames IA 50011 USA

## Abstract

The *naked endosperm1* (*nkd1*), *naked endosperm2* (*nkd2*), and *thick aleurone1* (*thk1*) genes are important regulators of maize (*Zea mays* L.) endosperm development. Double mutants of *nkd1* and *nkd2* (*nkd1,2*) show multiple aleurone (AL) cell layers with disrupted AL cell differentiation, whereas mutants of *thk1* cause multiple cell layers of fully differentiated AL cells. Here, we conducted a comparative analysis of *nkd1,2* and *thk1* mutant endosperm transcriptomes to study how these factors regulate gene networks to control AL layer specification and cell differentiation. Weighted gene coexpression network analysis was incorporated with published laser capture microdissected transcriptome datasets to identify a coexpression module associated with AL development. In this module, both *Nkd1,2+* and *Thk1+* appear to regulate cell cycle and division, whereas *Nkd1,2+*, but not *Thk1+*, regulate auxin signaling. Further investigation of *nkd1,2* differentially expressed genes combined with published putative targets of auxin response factors (ARFs) identified 61 AL‐preferential genes that may be directly activated by NKD‐modulated ARFs. All 61 genes were upregulated in *nkd1,2* mutant and the enriched Gene Ontology terms suggested that they are associated with hormone crosstalk, lipid metabolism, and developmental growth. Expression of a transgenic DR5–red fluorescent protein auxin reporter was significantly higher in *nkd1,2* mutant endosperm than in wild type, supporting the prediction that *Nkd1,2+* negatively regulate auxin signaling in developing AL. Overall, these results suggest that *Nkd1,2+* and *Thk1+* may normally restrict AL development to a single cell layer by limiting cell division, and that *Nkd1,2+* restrict auxin signaling in the AL to maintain normal cell patterning and differentiation processes.

## Core Ideas

- Transcriptomes were analyzed of *thk1* and *nkd1,2* mutants involved in maize endosperm development.
- *thk1* and *nkd1,2* genes coregulate cell division processes to control aleurone cell layer number.
- *nkd1,2* genes specifically regulate auxin signaling pathways in aleurone cell differentiation.

**Abbreviations**
ABA: abscisic acid
AL: aleurone
ARF: auxin response factor
BR: brassinosteroid
DAP: days after pollination
DAP‐seq: DNA affinity purification sequencing
DE: differentially expressed
DEG: differentially expressed gene
FDR: false discovery rate
GO: Gene Ontology
IAA: indole‐3‐acetic acid
IDD: INDETERMINATE DOMAIN
LCM: laser capture microdissected
*ocl*
: *outer cell layer*
SE: starchy endosperm
TF: transcription factor
TPM: transcripts per million
WGCNA: weighted gene coexpression network analysis.

## 1 INTRODUCTION

Maize (*Zea mays* L.) endosperm is the triploid product of double fertilization and provides nutrients for developing embryos and germinating seedlings. Endosperm is an important source of calories for humans and livestock and is also used as an industrial commodity. Early maize endosperm development undergoes three stages: (a) the coenocytic stage (1–3 d after pollination [DAP]), when the endosperm performs nuclear mitotic divisions without cytokinesis to produce a multinucleate coenocyte; (b) cellularization (3–4 DAP), when cell walls form between nuclei and subsequent cell division starts; and (c) differentiation (after 4 DAP), when major cell types are formed, including aleurone (AL), basal endosperm transfer layer, basal intermediate zone, conducting zone, embryo surrounding region, subaleurone, and starchy endosperm (SE) (Leroux et al., 2014; Sabelli & Larkins, 2009).

In most maize lines, AL is a single, epidermis‐like, cell layer of the endosperm (Becraft & Asuncion‐Crabb, 2000). It features abundant lipid bodies and thickened cell walls (Kyle & Styles, 1977; Zheng & Wang, 2014). Regulated by a balance of gibberellic acid and abscisic acid (ABA) signaling, AL cells produce amylases during germination to digest storage materials in SE for seedling growth (Hoecker et al., 1999). AL also contains abundant indigestible fibers, antioxidants, ferulic acid, and minerals beneficial for health (Lillioja et al., 2013).

The cellularization stage begins as anticlinal cell walls form between nuclei arranged around the periphery of the coenocyte. Then periclinal divisions generate a cellular peripheral layer and an alveolar interior layer (Olsen, 2001). Cell division in the peripheral layer is highly ordered with division planes oriented exclusively in either the anticlinal plane, to expand the number of cells in the peripheral layer and accommodate endosperm growth, or in the periclinal plane, to contribute new daughter cells to the endosperm interior (Becraft & Yi, 2011; Olsen, 2001). The highly ordered peripheral cells develop as AL, whereas the interior cells have random division planes and develop as subaleurone and SE (Becraft & Yi, 2011; Olsen, 2001).

AL development is controlled by several gene networks and signaling pathways (summarized in Supplemental Figure S1). The *defective kernel 1* (*dek1*) gene encodes a transmembrane protein functioning as a mechanosensitive calcium channel with a cytosolic calpain cysteine proteinase domain (Lid et al., 2002; Tran et al., 2017; Wang et al., 2003). Loss of function *dek1* mutants completely lack AL, indicating DEK1 is required as a positive regulator of AL cell fate (Becraft et al., 2002; Lid et al., 2002). When *dek1* mutants were induced somatically at later stages of development in a normal endosperm background, AL cells lost their identity and transdifferentiated to SE cells. Conversely, late‐stage somatic gene reversion of *dek1* mutant to wild type (WT) in the outermost cell layer of endosperm caused transdifferentiation of SE to AL identity. This highlighted the plasticity of AL cell fate and indicated that positional signaling pathways regulated by DEK1 were active throughout endosperm development (Becraft & Asuncion‐Crabb, 2000; Becraft & Yi, 2011). Another transmembrane protein, CRINKLY4 (CR4), mediates growth factor‐like responses associated with cell proliferation and differentiation of epidermal cells (Becraft et al., 1996; Jin et al., 2000). In *cr4* mutants, AL is absent in the abgerminal region, indicating CR4 is another positive regulator of AL cell fate (Becraft et al., 1996; Jin et al., 2000). *Supernumerary aleurone 1* (*sal1*) encodes a class E vacuolar sorting protein, similar to human CHMP1 (Shen et al., 2003). In *sal1* mutants, endosperm has multiple AL layers, suggesting that SAL1 is a negative regulator of AL cell fate. SAL1 colocalizes with DEK1 and CR4 in endocytic vesicles, suggesting that SAL1 may negatively regulate DEK1 and CR4 by internalization and protein sorting activity (Tian et al., 2007).

**Core Ideas**

- Transcriptomes were analyzed of *thk1* and *nkd1,2* mutants involved in maize endosperm development.
- *thk1* and *nkd1,2* genes coregulate cell division processes to control aleurone cell layer number.
- *nkd1,2* genes specifically regulate auxin signaling pathways in aleurone cell differentiation.

The *thk1* gene is another negative regulator of AL cell fate and mutants cause multiple layers instead of the normal single layer of AL cells (Yi et al., 2011). The *thk1* gene encodes a homolog of NOT1, a scaffold protein of the CCR4‐NOT complex, a multifunctional regulatory complex that regulates cellular activities through various mechanisms including promoting transcript turnover by deadenylation (Villanyi & Collart, 2015; Wu et al., 2020). Transcriptomic analysis of *thk1* mutant endosperm showed that *Thk1+* may regulate cell division, hormone signaling, and stress responses (Wu et al., 2020). Interestingly, *thk1* mutants are epistatic to *dek1* suggesting that *Thk1+* functions downstream of *Dek1+* in a signaling pathway that controls AL cell fate (Yi et al., 2011). The molecular mechanism of this signaling interaction remains obscure.

NKD1 and NKD2 are zinc finger transcription factors (TFs) of the INDETERMINATE DOMAIN (IDD) family, encoded by syntenic duplicated genes, *nkd1* and *nkd2* (Yi et al., 2015). Double mutants of *nkd1* and *nkd2* (*nkd1,2*) show multiple layers of partially differentiated AL cells, showing that *Nkd1,2+* are required to restrict the number of AL cell layers as well as to promote the differentiation of AL cell characteristics (Yi et al., 2015). *Nkd1,2+* functions are reminiscent of Arabidopsis IDD TFs, JACKDAW (JKD) and BALDIBIS (BIB). These bind to SCARECROW (SCR) and SCARECROW‐LIKE23 (SCL23), functioning to restrict movement of the cell fate determinant SHORT‐ROOT (SHR), resulting in a single cell layer of endodermis (Long, Goedhart, et al., 2015; Long, Smet, et al., 2015). Transcriptomic analysis showed that *Nkd1,2+* regulate genes associated with cell division, cell differentiation, hormone signaling, starch biosynthesis, and nutrient storage, indicating a complex regulatory network for AL development (Gontarek et al., 2016).

Results suggest that *Thk1+* might interact with *Nkd1,2+* to regulate endosperm and AL development (Wu et al., 2020). Triple mutants of *nkd1,2* and *thk1* showed more AL‐like cell layers than either the *nkd1,2* or *thk1* mutant alone, suggesting additivity in regulating the number of AL layers. However, the cells showed the partially differentiated AL characteristics of the *nkd1,2* mutant indicating that *nkd1,2* was epistatic to *thk1* with regard to AL cell differentiation (Yi et al., 2011). This suggests that *Nkd1,2+* and *Thk1+* function through independent pathways to restrict AL cell fate to a single cell layer and that *Nkd1,2+* functions in AL cell differentiation downstream of *Thk1+*.

In this study, our goal was to dissect the relationships among *Nkd1,2+* and *Thk1+* functions by performing a comparative analysis of *nkd1,2* and *thk1* mutant transcriptomes. Weighted gene coexpression network analysis (WGCNA) revealed the overlapping functions of *Thk1+* and *Nkd1,2+* in controlling AL cell layer number and AL cell differentiation. We identified auxin signaling, mainly regulated by *Nkd1,2+*, as an important element in AL differentiation and the differential expression of a DR5–red fluorescent protein (RFP) auxin reporter between WT and *nkd1,2* mutant kernels supported this prediction.

## 2 MATERIALS AND METHODS

### 2.1 Plant materials

*nkd1‐Ds* and *nkd2‐0766* mutants were obtained from the Ac/Ds project (Ahern et al., 2009) and were backcrossed to W22 inbred for three generations. Homozygous WT, *nkd1‐Ds* (*nkd1*), or *nkd2‐0766* (*nkd2*) single mutants and *nkd1,2* double mutants were derived by self‐pollinating a *nkd1‐Ds*/+, *nkd2‐0766*/+ heterozygote and propagating F_2_ individuals of the appropriate genotypes. All materials for RNA isolation were grown in the field at the Curtiss Research Farm, Iowa State University, Ames, IA, during 2019. Sixteen DAP endosperm samples were collected from four independent ears of each genotype for RNA extraction.

The DR5–RFP transgenic maize line was obtained from David Jackson (Cold Spring Harbor Laboratory) and contains ER localized RFP under the regulation of the synthetic auxin‐responsive DR5 promoter (Gallavotti et al., 2008). The transgene was backcrossed to the B73 inbred four generations and then crossed to the *nkd1, nkd2* double mutant, also in a B73 background. The F_1_ plants were self‐pollinated to generate ears segregating all three factors, DR5–RFP, *nkd1*, and *nkd2*.

### 2.2 RNA extraction and sequencing

Total RNA was extracted following the published procedure (Li et al., 2014) except that GeneJET RNA Cleanup and Concentration Micro Kit (Thermo Fisher Scientific) was used to clean up DNaseI‐treated RNA. RNA quality was tested by AATI Fragment Analyzer (Agilent) at the DNA Facility, Iowa State University. RNA samples were shipped to the Health Sciences Center, University of Utah (Salt Lake City, UT) for library preparation with Illumina TruSeq RNA Library Prep Kit (Illumina) and 150‐bp paired‐end sequencing using NovaSeq 6000 with the S4 cell.

### 2.3 Transcriptomic data processing

FastQC (https://www.bioinformatics.babraham.ac.uk/projects/fastqc/) was performed for quality control of raw reads. The raw reads were mapped to B73 RefGen_v4 (AGPv4) by HISAT2 with default settings specific for paired‐end reads (v2.1.0) (Jiao et al., 2017; Kim et al., 2015). The mapped transcripts in output files were sorted by SAMtools (v1.9) (Li et al., 2009) and were assembled and normalized with StringTie (v1.3.6) (Pertea et al., 2016), using the maize genome annotation file Zea_mays.B73_RefGen_v4.43.gtf downloaded from Gramene (http://www.gramene.org/). Readcounts were calculated and analyzed by HTSEquation (v0.11.1) (Anders et al., 2015) and DEseq2 (v1.26.0) (Love et al., 2014). Differentially expressed genes (DEGs) were called using a generalized linear model‐based approach with the criteria |log2FC| >1 and false discovery rate (FDR) <0.05.

### 2.4 Coexpression network analysis

Transcriptome datasets of laser capture microdissected (LCM) *nkd1,2* (National Center for Biotechnology Information Gene Expression Omnibus [NCBI GEO] accession number: GSE61057) and *thk1* (NCBI GEO accession number: GSE155296) were collected from published studies (Gontarek et al., 2016; Wu et al., 2020). (Accession number of transcriptome datasets are listed in Supplemental Table S2, and accession number of genes are listed in Supplemental datasets.) The data were processed following the procedures described above. Whole endosperm transcriptome data of *nkd1,2* from this study were combined with *thk1* data to perform WGCNA. Batch effect was removed via the ComBat algorithm in the R package SVA (Johnson et al., 2007; Leek et al., 2012). Log2 transformed transcripts per million (TPM) data were filtered and non‐DEGs (with low TPM variation) across all genotypes were excluded. The WGCNA was performed according to published procedures (Langfelder & Horvath, 2007, 2008). Soft‐thresholding power 18 was chosen to build a signed network with the scale‐free topology. Merge threshold and minimum module size were set as 0.25 and 50, respectively. The network was visualized by Cytoscape 3.7.2 with a connectivity threshold of 0.06 (Shannon et al., 2003).

### 2.5 ARF DAP‐seq target analysis

Putative target genes of ARF13 (Zm00001d049295), ARF29 (Zm00001d026540), ARF34 (Zm00001d031064) and ARF35 (Zm00001d014690) were obtained from published ARF DNA affinity purification sequencing (DAP‐seq) peak datasets (Galli et al., 2018). Peaks located at 5′ UTR + 1 kbp upstream of the transcription start site were selected. The selected genes were overlapped with coexpression modules, DEGs of *nkd1,2*, *thk1*, and AL or SE preferential genes to investigate putative *Nkd1,2* or *Thk1+* modulated ARF targets.

### 2.6 GO enrichment analysis

Gene Ontology (GO) term enrichment analysis was performed at AgriGOv2 (http://systemsbiology.cau.edu.cn/agriGOv2/) with FDR <0.05 (Tian et al., 2017). Maize‐GAMER (AGPv4) dataset was used as the annotated background (Wimalanathan et al., 2018).

### 2.7 Hypergeometric test

Hypergeometric tests were performed as described (Zhan et al., 2015) to statistically evaluate whether two gene sets are significantly related by calculating the number of overlapping genes relative to the total number of protein‐coding genes (39,324) in the B73 genome (Jiao et al., 2017). The overlaps among datasets were visualized as heatmaps that were built by the R package gplots (https://github.com/talgalili/gplots).

### 2.8 Fluorescence microscopy and RFP quantification

Kernels were collected from three independent ears, each segregating *nkd1*, *nkd2*, and the DR5–RFP reporter. At least four mature kernels per phenotype (either WT or *nkd1,2*) per ear were collected, genotyped, and used to quantify RFP expression (fluorescence). Genotyping primers are as follows: *nkd1* (forward: CCGATCATGTATAGCATTTCTTCTT; reverse: GCTTCTTGATCCCCGTCAG; followed by sequencing the PCR products for detection of the SNP TGCAC → TGTAC [Yi et al., 2015]), *nkd2* (forward: CCAACGGCCACACGTATAGAACG; reverse: CAACAAATCGACAGGGAGCCGAG; the mutants should give no band) and DR5–RFP (forward: CCAAGCTGAAGGTGACCAA; reverse: TCTTCTTCTGCATTACGGGG [Gallavotti et al., 2008]; and the transgene should give a 288‐bp band). Then the kernels were hand‐sectioned at the abgerminal region and observed under an Olympus BX60 epifluorescence microscope equipped with a Chroma mCherry filter set (excitation 560 nm, dichroic barrier filter 600 nm, emission 635 nm). All images were captured under the 10× objective with a Jenoptic C‐5 camera set to 500‐ms exposure time and using constant gain settings. The fluorescence intensities were quantified by ImageJ (https://imagej.nih.gov/ij/index.html). Fluorescence intensity between WT and *nkd1,2* kernels for each ear was compared by Student's *t* test through JMP (pro14, SAS Institute). The pooled fluorescence intensity was compared via two‐way analysis of variance (ANOVA) with Type III sums of squares (SAS v9.4, SAS Institute).

Confocal fluorescence microscopy was performed by the Roy J. Carver High Resolution Microscopy Facility, Iowa State University. The WT and *nkd1,2* kernels were collected at 30 DAP from the same segregating ears expressing the DR5–RFP transgene. The kernels were hand‐sectioned and mounted in phosphate buffered saline solution under a coverslip on the confocal microscope (Leica SP5 X MP). Images were captured under the 20× objective with pinhole size set at 1 and 39% laser power. Excitation and emission parameters for red fluorescence were set at 555 nm and a range of 581–708 nm, respectively. Images from bright field and red fluorescence channels were merged by Fiji‐ImageJ v1.53c.

## 3 RESULTS

### 3.1 Comparative analysis of *thk1* and *nkd1,2* mutant transcriptomes

Both *thk1* and *nkd1,2* mutants cause multiple AL layers, whereas *nkd1,2* mutants also disrupt AL differentiation. We hypothesized that a comparative transcriptomic analysis of these mutants would allow the identification of genes that control the specification of AL layer number versus genes involved in AL cell differentiation. A transcriptomic analysis of *thk1* mutant endosperm was recently published (Wu et al., 2020). This consisted of three genotypes: WT and two mutant alleles, *thk1‐iso15* and *thk1‐iso17*. Although a transcriptomic analysis of LCM *nkd1,2* mutant endosperm was previously published (Yi et al., 2015), we conducted a new analysis on *nkd1‐Ds* and *nkd2‐0766* null mutant alleles using samples and methods more comparable to the *thk1* data. Sixteen DAP endosperms were collected from WT, *nkd1‐Ds* and *nkd2‐0766* single mutants, and *nkd1,2* double mutant, and RNA was isolated and subjected to 150 base paired‐end RNA sequencing. An average of 36,928,566, 38,984,745, 40,343,136, and 50,055,590 unique reads were aligned to B74 v4 reference genome for *WT, nkd1‐Ds, nkd2‐0766*, and *nkd1,2*, respectively, with read alignment at least 71%. Genes were declared differentially expressed (DE) if they showed at least a twofold difference in expression between WT and mutant with adjusted *p*‐value of .05. In *nkd1,2* mutants, there were 2,504 genes upregulated and 1,389 genes downregulated; and in *thk1* mutants, there were 1,194 genes upregulated and 405 genes downregulated (Wu et al., 2020) (Supplemental Figure S2). There were 452 DEGs (9% of total DEGs) overlapping between the two datasets. Gene Ontology term enrichment analysis showed that the overlapping DEGs are associated with cell wall organization or biogenesis, flavonoid biosynthesis, auxin signaling, and transmembrane transport. Differentially expressed genes specific to *thk1* are mainly associated with cell division regulation, whereas DEGs specific to *nkd1,2* are mainly associated with cell differentiation regulation, auxin signaling, carbohydrate metabolism, and lipid storage (Supplemental Figure S2).

### 3.2 Coexpression network analysis of *thk1* and *nkd1,2* mutants

The DEG GO term results suggest that *Thk1+* and *Nkd1,2+* may coregulate some gene networks, while regulating other networks independently. To further dissect their respective gene networks, WGCNA (Langfelder & Horvath, 2008) was performed based on the combined TPM data described above. Six datasets analyzed consisted of (a) *Thk1+* WT, (b) *thk1* mutants *thk1*‐*iso15* and *thk1‐iso17*, (c) *Nkd+* WT, (d) *nkd1‐Ds* single mutant, (e) *nkd2‐0766* single mutants, and (f) *nkd1,2* double mutant. The data were log2 transformed by log2(TPM+1) and batch effects were removed via ComBat (Johnson et al., 2007; Leek et al., 2012). The results showed that row means (average expression of a gene across all genotypes) are highly correlated (*R*_before_^2 ^= 0.38, *p *<1 × 10^−200^ vs. *R*_after_^2^ **= 0.99, *p *<1 × 10^−200^) between *thk1* WT/mutant and *nkd* WT/mutant datasets, indicating that the datasets are comparable and suitable to perform WGCNA (Supplemental Figure 3a, b). The combined data were filtered to exclude non‐DEGs across all genotypes and a total of 5,735 genes were obtained to proceed with subsequent analyses.

**FIGURE 1:**
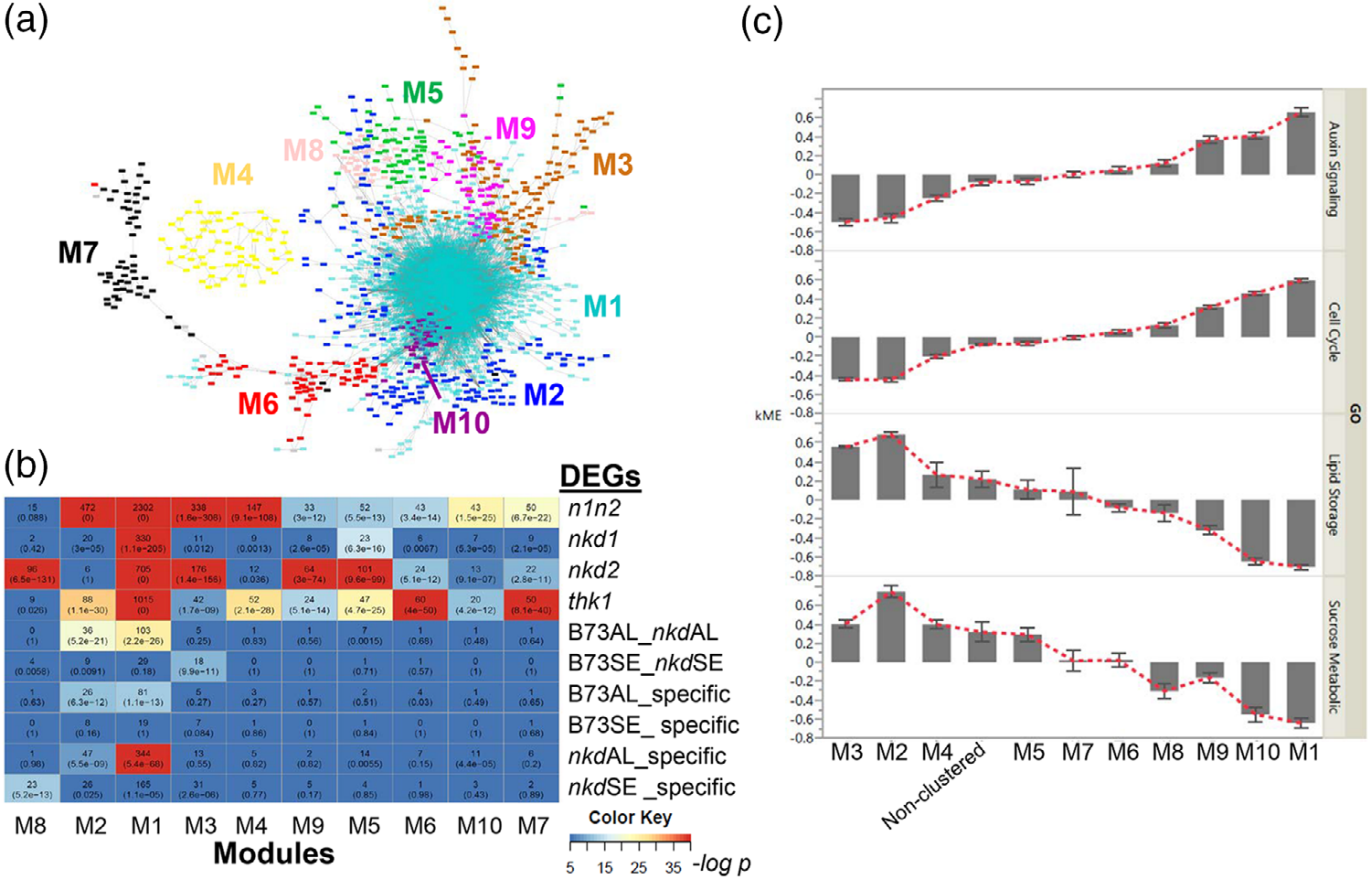
Coexpression network analysis of *nkd1,2* and *thk1* transcriptomes. (a) Network layout of 10 coexpression modules, color‐coded. (b) Overlap between module‐specific genes and differentially expressed genes (DEGs) of *nkd1* or *nkd2* single mutant (nkd1 and nkd2, respectively), *nkd1,2* double mutant (n1n2), *nkd1,2* AL tissue by laser capture microdissected (LCM) (nkd_AL), *nkd1,2* starchy endosperm (SE) tissue by LCM (nkd_SE), and *thk1* mutant (thk1). The top number in each cell represents the number of overlapping genes and the bottom parenthesized number the corresponding *p*‐value determined by hypergeometric test. The modules are reordered and clustered by Euclidean distance of columns. (c) Average module memberships (kME) for genes associated with corresponding Gene Ontology (GO) terms across all modules. The modules are reordered by kME

The WGCNA showed that all but 445 genes were clustered into 10 coexpression modules (networks) coded by M1 through M10 (Figure 1a) in descending order of the number of genes contained. Modules ranged from 3,551 genes in M1 to 71 genes in M10. All filtered genes from each genotype were clustered according to correlations with modules (Supplemental Figure 3c). Genes in the *nkd1,2* dataset (n1n2_whole) are positively correlated with M1 and negatively correlated with modules M2, M3, and M4, whereas genes in the *thk1* dataset (thk1_iso15 and thk1_iso17) are positively correlated with M1, M6, and M7 (Supplemental Figure 3c). That both *nkd1,2* and *thk1* datasets are correlated with M1 indicates that *Nkd1,2+* and *Thk1+* might coregulate this module.

To investigate the contribution of each factor to the regulation of each module, hypergeometric tests (Zhan et al., 2015) were performed to calculate whether the number of overlapping DEGs between each genotype and module was greater than expected by random chance. The result showed that DEGs of *nkd1* or *nkd2* single mutants, *nkd1,2* double mutant, and *thk1* mutant are strongly associated with M1 (Figure 1b). Highly significant overlaps were also observed among DEGs of *nkd1,2* with modules M2, M3, and M4 and among *thk1* DEGs with modules M6 and M7 (Figure 1b). To examine the relationship between *nkd1,2* and related modules with respect to tissue types, the previously published RNA‐seq datasets of LCM AL and SE of *nkd1,2* mutant were incorporated in the test (Gontarek et al., 2016). The raw reads were realigned to B73v4 reference genome and DEGs of B73AL vs B73SE and *nkd*AL vs *nkd*SE were called. The upregulated genes represent genes preferentially expressed in AL of WT and *nkd1,2* backgrounds, respectively, while downregulated genes represent genes preferentially expressed in SE (Supplemental Figure S4a and b). Specific to the WT background, there were 375 genes preferentially expressed in AL and 404 in SE (Supplemental Figure S4b, regions a and b, respectively). Specific to the *nkd1,2* mutant background, there were 1,414 and 1,319 genes preferentially expressed in AL and SE, respectively (Supplemental Figure 4b, regions c and d). There are 364 AL‐preferential and 269 SE‐preferential genes shared in both WT and *nkd* backgrounds (Supplemental Figure 4b, regions g and h). Very few genes alter their tissue preference between the WT and *nkd1,2* background (Supplemental Figure 4b, regions e and f). The hypergeometric test showed that AL‐preferential genes in both WT and *nkd1,2* backgrounds have significant overlap with M1 and M2 (Figure 1b), indicating that these two modules may be associated with AL development. Gene Ontology term enrichment analysis showed that M1 is associated with cell cycle regulation and hormone signaling, especially auxin signaling (Table 1). The module M3 is associated with heat response, whereas M2 is associated with sucrose metabolism and lipid storage. No enriched GO terms were detected for modules M4, M6, or M7.

**TABLE 1:** Major Gene Ontology (GO)‐terms and corresponding false discovery rates (FDRs) in modules and categories

**GO_ID**	**Description**	**FDR**	**Module or category**
GO:0009734	Auxin‐activated signaling pathway	2.61E‐05	M1
GO:0007049	Cell cycle	6.34E‐04	M1
GO:0005985	Sucrose metabolic process	1.29E‐02	M2
GO:0019915	Lipid storage	1.30E‐03	M2
GO:0009408	Response to heat	2.10E‐35	M3
GO:0042759	Long‐chain fatty acid biosynthetic process	1.30E‐04	M5
GO:0000911	Cytokinesis by cell plate formation	2.3E‐04	Group I
GO:0000911	Cytokinesis by cell plate formation	1.4E‐14	Group II
GO:0009734	Auxin‐activated signaling pathway	5.6E‐03	Group III
GO:0007389	Pattern specification process	5.5E‐03	Group III
GO:0009741	Response to brassinosteroid	3.7E‐03	ARF_A, *nkd1,2–*DEGs
GO:0060560	Developmental growth involved in morphogenesis	2.6E‐02	ARF_A, *nkd1,2–*DEGs
GO:0009733	Response to auxin	3.7E‐03	ARF_A, *nkd1,2–*DEGs

*Note*. ARF‐A, Clade A auxin response factor; DEGs, differentially expressed genes; *nkd1,2*, double mutants of *naked endosperm1* and *naked endosperm2*.

Eigengene‐based connectivities, or module memberships (kME), of GO term‐associated genes in each module represent correlations of each gene or group of genes with the module eigengenes (Langfelder & Horvath, 2007, 2008). The higher the kME value in a module, the higher correlation of the gene(s) with the corresponding module. The kMEs of genes associated with selected GO terms were calculated across each module and auxin signaling and cell cycle showed the highest kMEs for M1 (Figure 1c), consistent with the GO term–module relationship reported in Table 1. Taken together, these results suggest that *Thk1+* and *Nkd1,2+* coregulate M1, which is important for AL development, and that cell cycle regulation and auxin signaling may be important aspects of this process. Lipid storage and sucrose metabolism, showed the highest kMEs in M2 (Figure 1c), suggesting that this module is important for *Thk1+* and *Nkd1,2+* regulation of storage product metabolism in AL.

### 3.3 Gene expression modules regulating AL development

**FIGURE 2:**
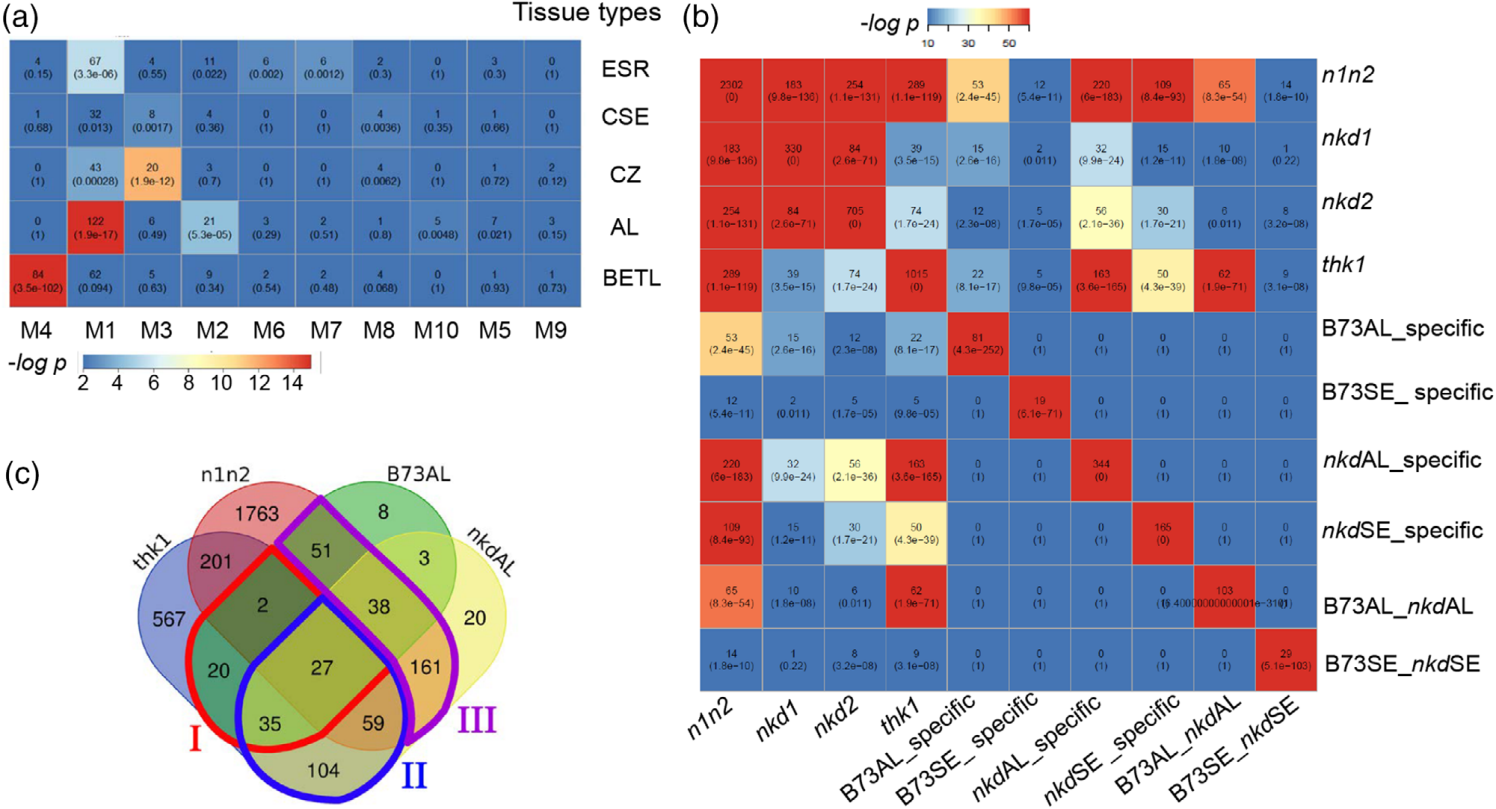
Analysis of module M1 associated with aleurone (AL) development. (a) Overlap between module‐specific genes and tissue specific genes. The modules are reordered and clustered by Euclidean distance of columns. (b) Overlap between differentially expressed genes (DEGs) of *nkd1,2* double mutant, *nkd1* or *nkd2* single mutants, *thk1* mutant, and AL or starchy endosperm (SE)‐preferential genes in wild type (WT) or *nkd* background within M1. For (a) and (b), the top number in each cell represents the number of overlapping genes and the bottom parenthesized number the corresponding *p*‐values by hypergeometric test. (c) Venn diagram showing overlap between DEGs of *thk1*, *nkd1,2*, (n1n2), and AL‐preferential genes in WT (B73AL) and *nkd1,2* (nkdAL) backgrounds within M1

To analyze relationships between endosperm tissues and coexpression modules, hypergeometric tests were performed to investigate overlapping genes in the modules from this study with previously published endosperm tissue‐specific LCM RNA‐seq data (Zhan et al., 2015). The results showed that genes expressed in developing AL were significantly overrepresented in M1 and, to a lesser extent, in M2 (Figure 2a). This is consistent with Figure 1b, where genes preferentially expressed in AL significantly overlap modules M1 and M2. In addition to AL, M1 also overlapped with conducting zone and embryo surrounding region (Figure 2a). Developing conducting zone and basal endosperm transfer layer genes also significantly overlapped with modules M3 and M4, respectively (Figure 2a). These results suggest that these coexpression modules may be involved in tissue‐specific regulation of endosperm development.

Within M1, *thk1* and *nkd1,2* DEGs have more significant overlap with AL‐preferential genes than SE‐preferential genes in both WT and *nkd* backgrounds (Figure 2b), indicating that *Thk1+* and *Nkd1,2+* regulate several biological processes in common that function in developing AL. DEGs of *nkd1* or *nkd2* single mutants have strong overlap between one another and with *nkd1,2* double mutant; however, compared with the double mutant, the single mutants have less significant overlap with AL‐preferential genes (Figure 2b). This suggests that *Nkd1+* and *Nkd2+* have unique functions as well as redundant functions, consistent with the partial redundancy reported previously (Yi et al., 2015).

To further explore the functional relationships amongst genes within M1, the overlap was examined among DEGs of *thk1*, *nkd1,2*, and AL‐preferential genes in WT and *nkd* backgrounds (Figure 2c). Three groups of particular interest are highlighted. Group I (84 genes) represents AL‐preferential genes from a WT B73 background that are regulated by *thk1*. The GO term *cytokinesis by cell plate formation* (GO:0000911, FDR = 0.00023) was enriched (Table 1) in this group suggesting that *thk1* may regulate mitotic cytokinesis during AL development. Group II (225 genes) represents AL‐preferential genes from a *nkd1,2* mutant background that may also be regulated by *thk1*. The same GO term as Group I was enriched with higher FDR (1.4 × 10^−14^) (Table 1), suggesting both *thk1* and *nkd1,2* may be involved in controlling cell division processes in developing AL. Group III (250 genes) represents AL‐preferential genes from either WT or *nkd* mutant backgrounds that are regulated by *nkd1,2* but not by *thk1*. The GO terms *auxin‐activated signaling pathway* (GO:0009734, FDR = 0.0056) and *pattern specification process* (GO:0007389, FDR = 0.0055) were enriched, suggesting that *nkd1,2* has specific functions in regulating auxin signaling and cell patterning during AL development and these are not shared by *thk1*.

### 3.4 *Nkd1,2+* regulate ARFs in developing AL

Responses to auxin signaling are regulated in large part by auxin response factors (ARFs), TFs that activate or repress expression of auxin‐regulated genes and control multiple developmental processes in plant tissues (Guilfoyle & Hagen, 2007; Leyser, 2018). Phylogenetic analysis of the ARF family identifies three clades (Galli et al., 2018). Transcription reporter assays showed that most Clade A ARFs (ARF‐A) are transcriptional activators; most Clade B ARFs (ARF‐B) are transcriptional repressors; and most Clade C ARFs (ARF‐C) produced no change in target gene expression (Galli et al., 2018).

**FIGURE 3:**
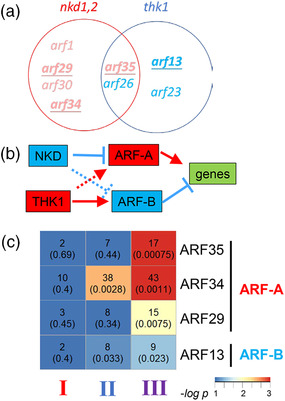
Analysis of *Nkd1,2+*‐ or *Thk1+*‐modulated ARF genes in aleurone (AL) development. (a) Auxin response factor (ARF) genes that were differentially expressed (DE) in *nkd1,2* or *thk1* mutants. Pink‐ and light blue‐labeled genes represent Clade A ARF (ARF‐A) and Clade B ARF (ARF‐B) genes, respectively. Red and blue circles represent upregulated and downregulated genes, respectively. The underlined genes encode proteins with published DNA affinity purification sequencing (DAP)‐seq target data available. (b) Model for the transcriptional relationships among *Nkd1,2+*, *Thk1+*, ARF‐A, ARF‐B, and downstream genes. Red and blue‐highlighted factors represent activators and repressors, respectively. (c) Overlap between ARF putative target genes and AL‐preferential gene Groups I, II, and III from Figure 2c. Top numbers in each cell represent the number of overlapping genes with corresponding *p*‐values by hypergeometric test in parentheses

Eight ARF genes were DE in *nkd1,2* and/or *thk1* mutants. Interestingly, all ARFs that were DE in *nkd1,2* were upregulated, and most of them are ARF‐A, whereas all ARFs DE in *thk1* were downregulated, and most of them are ARF‐B (Figure 3a). This suggests that *Nkd1,2+* mainly repress the expression of ARF‐A, and *Thk1+* mainly activates expression of ARF‐B (Figure 3b). Among the *nkd1,2*‐regulated ARF genes, *arf35* contains NKD1 and NKD2 binding motifs in *cis*‐regions, whereas *arf26* and *arf29* contain NKD1 *cis‐*binding motifs, identifying these as putative direct target genes of NKD1 and/or NKD2 regulation (Gontarek et al, 2016). Putative downstream target genes were also previously identified for several of the ARFs that are modulated by *nkd1,2* or *thk1* (ARF‐A: ARF29, 34 and 35; ARF‐B: ARF13). DNA affinity purification sequencing was used to identify *cis‐*binding sites for these ARFs on a genome‐wide scale (Galli et al., 2018). We examined the overlap between these putative ARF target genes with genes in coexpression modules, DEGs of *nkd* mutants, DEGs of *thk1* mutant, as well as AL and SE‐preferential genes. The results showed that ARF‐A target genes have strong overlap with M1, and ARF34 and ARF13 targets have lesser overlaps with M2 and M7 (Supplemental Figure 5a). Also, *nkd1,2* DEGs have strong overlap with selected ARF targets, whereas *nkd1* or *nkd2* single mutants do not (Supplemental Figure 5b), consistent with redundancy of *nkd1* and *nkd2* (Yi et al., 2015). There are 61 genes overlapping among ARF‐A putative targets, *nkd1,2* DEGs and AL‐preferential gene Groups I + II + III (Figure 2c and Supplemental Figure 6a). These 61 genes are most likely regulated by an NKD–ARF pathway associated with AL development. Differentially expressed genes of *thk*1 have significant overlap with targets of ARF34, 35, and 13 (Supplemental Figure S5b). Note that ARF34 is not DE in the *thk1* mutant (Figure 3a), suggesting these DEGs may be regulated independently of ARF34, or by a posttranscriptional mechanism. There are only two genes overlapping amongst putative targets of ARF13 and 35, *thk1* DEGs and the AL‐preferential gene Group I (Supplemental Figure 6b), indicating that the ARF signaling pathways may not play a major role in *thk1*‐mediated AL development. The 92 genes contained in both the ARF35 or 13 DAPseq peaks and *thk1* DEGs may be associated with functions that are not specific to AL.

To focus on how ARFs regulate AL development, overlap was examined between selected ARF target genes and AL‐preferential gene groups from Figure 2c. Group III (*nkd*‐regulated AL) genes most significantly overlapped with ARF‐A targets, Group II genes (*thk1‐* and *nkd*‐regulated AL) showed less significant overlap with ARF34 and ARF13, and there was no significant overlap between Group I (*thk1*‐regulated WT AL) and targets of any ARF (Figure 3c). These results suggest a close relationship between *nkd1,2*, ARF‐A, and AL development and are consistent with the enriched GO term *auxin‐activated signaling pathway* of Group III (Table 1).

**FIGURE 4:**
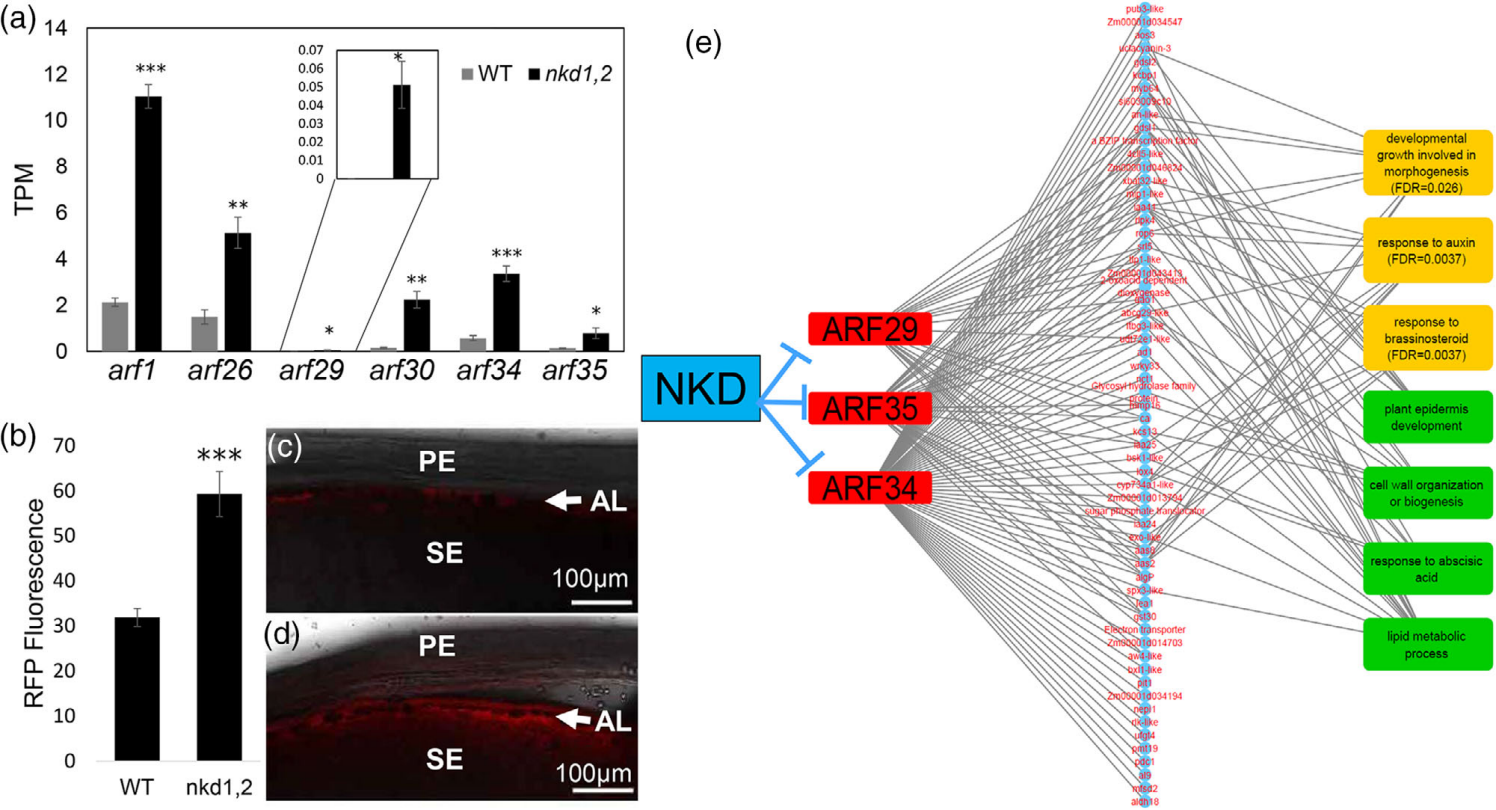
*Nkd1,2+* regulation of auxin signaling. (a) Expression comparison of auxin response factor (ARF) genes between wild type (WT) and *nkd1,2*. Normalized expression values were quantified by transcripts per million reads (TPM), and error bars represent standard errors. *, **, and *** represent *p*‐value <0.05, 0.01, and 0.0001, respectively, by *t*‐test. (b) Quantification of red florescent protein (RFP) fluorescence from microscopic images of WT and *nkd1,2* mutant kernels expressing DR5–red florescent protein (RFP). Shown are means of kernels sampled from 3 independent ears. ***p‐value <.001 by analysis of variance. Error bars represent standard errors. (c and d) Confocal fluorescence microscopy images of AL and adjacent regions in WT (c) and *nkd1,2* mutant (d) kernels expressing the DR5–RFP transgene. AL, aleurone; SE, starchy endosperm; PE, pericarp. Scale bar = 100 μm. (e) NKD‐modulated ARF‐A putative target genes, and corresponding GO terms. Yellow highlights are significantly enriched Gene Ontology (GO) terms, and GO terms highlighted in green were not significantly enriched but are biologically relevant. The 61 genes represent the intersection of putative Clade A auxin response factor (ARF‐A) targets, *nkd1,2* DEGs, and AL‐preferential gene Groups II + III. DEG, differentially expressed genes. (Refer to Supplemental datasets for gene_id and detailed information)

Differential expression of ARF genes in *nkd1,2* (Figure 4a) suggested that *Nkd1,2+* regulates auxin response. To test this prediction, a DR5–RFP transgene, with the synthetic auxin‐responsive DR5 promoter driving RFP (Gallavotti et al., 2008) was crossed to *nkd1,2* and expression studied in kernels from segregating F_2_ ears. Expression of this auxin reporter was significantly higher in *nkd1,2* mutant endosperm than in WT (Figure 4b, Supplemental Tables S1 and S2). In WT endosperm, RFP expression was most prominent in the AL cells (Figure 4c), whereas in *nkd1,2* mutant endosperm, expression was observed deeper into the endosperm tissues (Figure 4d). There was significant variation among ears sampled from different plants, but within each ear, *nkd1,2* showed significantly higher mean RFP fluorescence than WT (Supplemental Table S1, Supplemental Figure S7). These data suggest that *Nkd1,2+* function to restrict auxin response levels, as well as spatial distribution in the endosperm, and support the hypothesis that *Nkd1,2+* control ARF‐modulated auxin signaling in AL development.

To explore the biological processes that are regulated by NKD‐ARF‐mediated auxin signaling in AL development, GO enrichment analysis was conducted on the 61 genes at the intersection of ARF‐A putative targets, *nkd1,2* DEGs, and AL‐preferential genes (Supplemental Figure S6a). The results showed that these genes may be involved in developmental growth, auxin response, brassinosteroid (BR) response, and lipid metabolism, and, to a lesser extent, abscisic acid response, cell wall organization, and plant epidermis development (Figure 4e, Table 1). The 61 target genes of *nkd1,2‐*modulated ARF‐As are all upregulated in the *nkd1,2* mutant (Supplemental datasets), which supports the proposed model in Figure 3b that *Nkd1,2+* negatively regulate these genes through repression of ARF‐As. Many of these genes belong to multiple GO terms, suggesting that they may be involved in more than one biological process.

## 4 DISCUSSION

Genetic studies suggested that *Thk1+* and *Nkd1,2+* functions interact to regulate the number of AL cell layers, AL cell differentiation, and other aspects of endosperm tissue development (Gontarek et al., 2016; Yi et al., 2011; Yi et al., 2015). THK1 is a NOT1 homolog, a scaffolding subunit of the multifunctional CCR4‐NOT complex, whereas NKD1,2 are IDD family zinc finger TFs. As such, it is unclear how these regulatory functions are related. We sought to explore these relationships through comparative transcriptomics and gene network analysis of *thk1* and *nkd1,2* mutants. Nine percent of the total DEGs were DE in both mutants; these were enriched for functions associated with cell wall assembly, flavonoid biosynthesis, auxin signaling, and membrane transport. To better understand how the *Thk1+* and *Nkd1,2+* regulatory systems regulate these processes, WGCNA was performed using RNAseq data from mutants and corresponding WT, normalized from published studies (Gontarek et al., 2016; Yi et al., 2015) and additional RNA sequencing.

The RNAseq data used in the WGCNA study were derived from whole endosperm, composed of multiple tissues. To help determine tissue‐specific aspects of expression, we turned to published LCM studies (Gontarek et al., 2016; Zhan et al., 2015). Although these data could not be compared directly due to differences in developmental stages and sampling methods, the overlap between tissue‐specific genes and genes of our coexpression modules was informative. The coexpression module designated M1 was the largest module (Figure 1a). Module M1 had more significant overlap with AL specific or preferential genes than with SE or any other endosperm tissue at either 8 or 15 DAP (Figure 1b, 2a). That the largest coexpression module appears to be associated with developing AL is consistent with the pronounced AL phenotypes of *thk1* and *nkd1,2* mutants (Yi et al., 2011; Yi et al., 2015).

Two major GO terms, *cell cycle* (GO:0007049) and *auxin‐activated signaling pathway* (GO:0009734), were enriched in M1 (Table 1). The GO term *cytokinesis by cell plate formation* (GO:0000911) was enriched in genes of the overlap among DEGs of *thk1* and *nkd1,2*, contained in M1 and which were AL‐preferential (Figure 2c, Groups I and II; Table 1). The GO term *auxin‐activated signaling pathway* was enriched in Group III (Figure 2c), containing M1 genes that were AL‐preferential and DE in *nkd1,2* but not DE in *thk1*. Recall that *nkd1,2* and *thk1* both produced multiple‐AL‐layer mutant phenotypes, whereas *nkd1,2*, but not *thk1*, showed significant AL differentiation defects (Yi et al., 2011; Yi et al., 2015). As such, these results suggest that regulation of cell division might be associated with controlling AL cell layer number, whereas auxin‐activated signaling pathways and cell wall formation might be associated with *Nkd1,2+*‐regulated AL differentiation.

The gene expression profiles provide several clues to how *Nkd1,2+* and *Thk1+* may function to coordinate cell patterning and differentiation during endosperm development. Intriguingly, four GRAS family TFs (*gras2*, *gras29*, *gras33*, and *gras55*) were DE in *nkd1,2* mutants (Supplemental datasets). As reviewed by Kumar et al. (2019), GRAS family members interact with IDD family TFs in several systems to regulate cell division, cell patterning, cell specification, and hormone signaling. This includes the well‐known system in root cell patterning where the GRAS factors SHORTROOT and SCARECROW act in concert with IDD factors including JACKDAW to control the number and identity of cell layers (Long, Goedhart, et al., 2015; Long, Smet, et al., 2015). In addition, five GRAS family TFs (*gras7*, *gras37*, *gras38*, *gras42*, and *gras76*) were DE in the *thk1* mutant (Wu et al., 2020), but there was no overlap with *nkd1,2* suggesting that *Thk1+* and *Nkd1,2+* regulate independent GRAS pathways. Similarly, several homeodomain TF genes of the *outer cell layer* (*ocl*) family, including *ocl1*, *3*, *and 4* were DE in *nkd1,2* mutants and belong to module M1. In addition, *ocl4* is an AL‐preferential gene that is upregulated in *thk1* mutant (Gontarek et al., 2016; Wu et al., 2020) (Supplemental datasets). Altered cell division and patterning in developing anthers of maize *ocl4* mutants resulted in an extra subepidermal cell layer with endothecium characteristics, causing partial male sterility (Vernoud et al., 2009). Thus, it is reasonable to speculate that regulation of *ocl4* in endosperm may contribute to controlling the number of AL layers. The regulation of cell cycle and cell division is critical for cell patterning and tissue differentiation, and genes involved in cell cycle and cell division were DE in both *thk1* and *nkd1,2* mutants. Several cell cycle genes were AL‐preferential in *nkd1,2* background but were not DE in whole endosperm of *nkd1,2* mutants (Figure 2c, Supplemental datasets), suggesting that *Nkd1,2+* may preferentially regulate cell cycle and division in AL compared with other tissues. Prior studies showed that a significantly greater number of cell cycle genes were DE in AL than SE (Gontarek et al., 2016).

Auxin plays an essential role in plant growth and development, and ARFs are key mediators of auxin signaling and regulators of auxin response genes (Guilfoyle & Hagen, 2007; Li et al., 2016; Roosjen et al., 2018). Both *Nkd1,2+* and *Thk1+* appear to restrict expression of auxin response genes in maize endosperm but likely through different pathways: *Nkd1,2+* mainly inhibits genes for ARF‐As, activators of auxin response genes, whereas *Thk1+* mainly activates genes for ARF‐Bs, repressors of auxin response genes (Figure 3a, b). Downstream target genes containing *cis‐*binding sites for several of the DE ARFs were previously identified by DAP‐seq (Galli et al., 2018). These putative ARF target genes are more significantly enriched among genes preferentially expressed in AL than SE and have more overlap with AL‐preferential genes regulated by *Nkd1,2+* than by *Thk1+* (Supplemental Figure S5b, S6a and b). Furthermore, no GO terms were enriched among the *thk1* DEGs preferentially expressed in AL (Supplemental Figure S6b). This suggests that *Nkd1,2+* may have a more prominent function than *Thk1+* to regulate auxin signaling in AL development, which is consistent with the enriched GO term of *auxin‐activated signaling pathway* in AL‐preferential genes that are regulated by *nkd1,2* but not *thk1* (Figure 2c Group III; Table 1). Several putative targets of ARF34 or ARF13 are DE in *thk1* or *nkd1,2*, respectively, even though these ARF genes themselves are not DE in the corresponding mutant (Supplemental Figure S5b). These targets might be regulated by factors other than ARFs that are DE in the mutants, or the ARFs might be indirectly regulated by posttranscriptional mechanisms.

Prior studies have implicated auxin in regulating AL development in a manner consistent with our results presented here. Developing maize endosperm has been shown to accumulate free indole‐3‐acetic acid (IAA), specifically in the AL layer, and treating plants with the auxin transport inhibitor N‐1‐naphthylphthalamic acid caused elevated levels of IAA and the formation of multiple layers of altered AL cells (Forestan et al., 2010). In our study, we found that *nkd1,2* mutants contain elevated levels of ARF‐A gene expression and elevated levels of auxin signaling, as reflected by expression of the DR5–RFP auxin reporter, in the multiple layers of abnormally differentiated AL cells. These results suggest that auxin regulation is important for AL cell patterning and development and that *Nkd1,2+* repression of ARF‐A gene expression may restrict auxin signaling in the AL to maintain normal cell patterning and differentiation. Expression of ARFs could potentially be directly repressed by NKD1,2 binding to their promoters, as was previously predicted for *arf26*, *arf29*, and *arf35* (Gontarek et al., 2016), or could be indirectly affected as would be the case for all *thk1*‐regulated ARF genes given that THK1 is not a TF. One possibility is that indirect regulation could be via auxin metabolism or transport given that some ARFs contain auxin response elements in their promoters (Galli et al., 2018). As shown in Figure 4e, several aspects of AL development appear to be regulated by auxin and the NKD‐ARF network. Of particular note are cell growth and morphogenesis and cell wall organization or biogenesis. Auxin signaling is well known to promote cell wall loosening and cell expansion by modifications of cell wall composition (Majda & Robert, 2018). The thinner walls and irregular shape of *nkd1,2* mutant AL cells (Becraft & Yi, 2011; Yi et al., 2015) might be associated with excessive auxin signaling. Lipid metabolism and plant epidermis development are also noteworthy functions because lipid accumulation is a key aspect of AL cells and AL has been proposed to be homologous to the plant epidermis (Becraft & Yi, 2011).

Putative ARF targets that were DE in *nkd1,2* are associated with signaling systems of several hormones, including auxin, BR, and ABA. This suggests that *Nkd1,2+*‐ARF auxin signaling may mediate crosstalk with other phytohormones. NKD1,2‐ARF‐A regulates two homologs of GH3 (Zm00001d006753, *aas2*, and Zm00001d022017, *aas8*). GH3 genes are involved in IAA inactivation and are induced by both IAA and BR in Arabidopsis and rice (*Oryza s ativa* L.) (Goda et al., 2004; Zhang et al., 2015). In rice, OsARF19 activates expression of OsGH3‐5 and *BRASSINOSTEROID INSENSITIVE1* (*OsBRI1*), and overexpression of OsARF19 altered the expression of genes associated with auxin and BR signaling. This resulted in an increase in leaf adaxial cell division; therefore, it is reasonable to speculate that auxin–BR crosstalk could also influence maize endosperm tissue architecture. ABA signaling is critical for promoting maturation and quiescence in AL cells and ABA‐related genes, including *viviparous1* (*vp1*) and *responsive to aba17* (*rab17*), are specifically expressed in AL (Cao et al., 2007; Gontarek et al., 2016). VP1 is a TF required for ABA regulation of gene expression (Suzuki et al., 2003) and the *vp1* gene is directly regulated by NKD1,2 (Gontarek et al., 2016). Auxin crosstalks with ABA to regulate seed maturation as well as root development. Arabidopsis ARF10 and ARF16 regulate *ABSCISIC ACID INSENSITIVE 3* (*ABI3*), the ortholog of VP1, which triggers ABA‐induced seed maturation and dormancy (Liu et al., 2013). Also, auxin signaling enhances VP1‐mediated ABA response in maize roots (Suzuki et al., 2001). The networks reported here provide further examples of auxin–ABA crosstalk.

**FIGURE 5:**
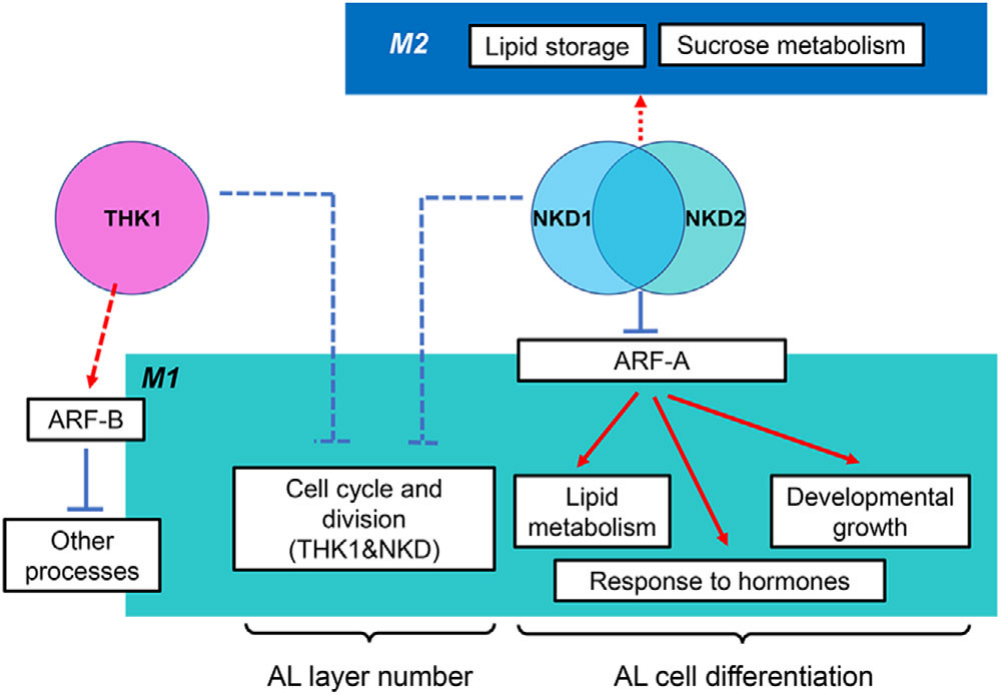
Model of *Nkd1,2+* and *Thk1+* regulation of AL development. *Nkd1,2+* and *Thk1+* appear to repress cell cycle and division, which may be important for endosperm cell patterning by limiting the number of aleurone (AL) layers. *Nkd1,2+* may promote AL differentiation by regulating developmental growth, hormone response and lipid metabolism via Clade A auxin response factor (ARF‐A)‐dependent pathways. Thk1+ may indirectly regulate ARF‐B but has limited impact on AL differentiation

In summary, Figure 5 illustrates a putative model regarding how *Nkd1,2+* and *Thk1+* networks regulate AL development. Both *Nkd1,2+* and *Thk1+* appear to limit the number of AL layers by restricting expression of cell cycle and cell division genes in different, partially overlapping, pathways. *Nkd1,2+* controls AL cell differentiation by regulating ARF‐A‐mediated plant cell morphogenesis, cell wall organization, hormone response and crosstalk, and lipid metabolism, whereas *Thk1+* may have a minor effect on AL cell features but may have other regulatory functions through ARF‐B. This study proposed a potential link between two types of AL regulators, *Nkd1,2+* and *Thk1+*, and identified coexpression modules (M1 and M2) that function in cell cycle, auxin‐activated signaling, and sugar/lipid metabolism, important for AL development.

## AUTHOR CONTRIBUTIONS

Hao Wu: Conceptualization; Formal analysis; Validation; Writing‐original draft; Writing‐review & editing. Philip W. Becraft: Conceptualization; Supervision; Writing‐review & editing.

## CONFLICT OF INTEREST

The authors declare no conflict of interest.

## Supporting information


**Supplemental** Table 1. Relative RFP intensity of mature DR5–RFP kernels between WT and *nkd1,2*
**Supplemental Table 2**. ANOVA output of RFP intensity between AL of WT and *nkd1,2* kernels.
**Supplemental Table 3**. Accession number of transcriptome datasets
**Supplemental** Figure 1. Major regulators of maize aleurone development and the relationships among them.
**Supplemental** 2. Venn diagram showing overlap between DEGs of *nkd1,2* and *thk1* datasets, and GO‐terms enriched in each region of overlap.
**Supplemental** Figure 3. Batch effect removal of WGCNA input data and network correlation with genes of each genotype.
**Supplemental** Figure 4. Differentially expressed genes between AL and SE by B73 WT and *nkd* backgrounds.
**Supplemental** Figure 5. Overlap between ARFs versus coexpression modules and DEGs.
**Supplemental Figure 6**. Overlap between ARF putative targets, AL preferential genes, and DEGs of *nkd1,2* or DEGS of *thk1*.
**Supplemental Figure 7**. Variation in fluorescence levels for the DR5–RFP transgene in 3 independent ears segregating WT and *nkd1,2* mutant kernels.

Supplemental Dataset 1. nkd1,2 double mutant DEGs

**Supplemental Dataset 2**. *nkd‐thk1* coexpression network modules and genes

Supplemental Dataset 3. Gene list of Group‐I, II and III in Figure 2C

**Supplemental Dataset 4**. Genes associated with cell division in Group‐I,

**Supplemental Dataset 5**. Genes associated with cell division in Group‐II

**Supplemental Dataset 6**. Genes associated with auxin signaling in Group‐III

**Supplemental Dataset 7**. AL‐preferential genes in B73 background

**Supplemental Dataset 8**. SE‐preferential genes in B73 background

**Supplemental Dataset 9**. AL‐preferential genes in *nkd1,2* background

**Supplemental Dataset 10**. SE‐preferential genes in *nkd1,2* background

**Supplemental Dataset 11**. ARF‐A putative target genes modulated by *Nkd1,2+*
