# Supplementary material for: Comparative transcriptomics and network analysis define gene coexpression modules that control maize aleurone development and auxin signaling
Source: Plant Genome. 2021 Jul 29;14(3):e20126. doi: 10.1002/tpg2.20126 (PMC12807064; doi:10.1002/tpg2.20126)
Supplement: Supplementary file 1 [file TPG2-14-e20126-s005.pdf]

SUPPLEMENTAL TABLES

Supplemental Table 1. Relative RFP intensity of mature DR5:RFP kernels between WT and *nkd1,2*

Ear_ID	Kernel phenotype	#kernel tested	Mean RFP Intensity	SE	*p**
DR5-1	WT	5	31.598	1.88	0.0003
	nkd1,2	5	71.924	9.89	
DR5-2	WT	11	36.298	3.41	0.0366
	nkd1,2	4	54.577	7.07	
DR5-3	WT	7	24.938	2.00	0.0372
	nkd1,2	5	50.537	10.72	

* *p*-values of mean differences by Student's *t* test

Supplemental Table 2. ANOVA output of RFP intensity between AL of WT and *nkd1,2* kernels.

Source	DF	Type III SS	Mean Square	F Value	Pr > F
**Phenotype**	1	6486.687163	6486.687163	42.33	<.0001***
**Ear_ID**	2	1096.295637	548.147818	3.58	0.0400
**Ear_ID-Phenotype**	2	648.203667	324.101833	2.12	0.1377

*** Significant difference by F-test of two-way ANOVA

Supplemental Table 3. Accession number of transcriptome datasets

Accession number	Description
GSE61057	Transcriptome datasets of LCM *nkd1,2*
GSE155296	Transcriptome datasets *thk1* whole endosperm
GSE174059	Transcriptome datasets of *nkd1,2* whole endosperm

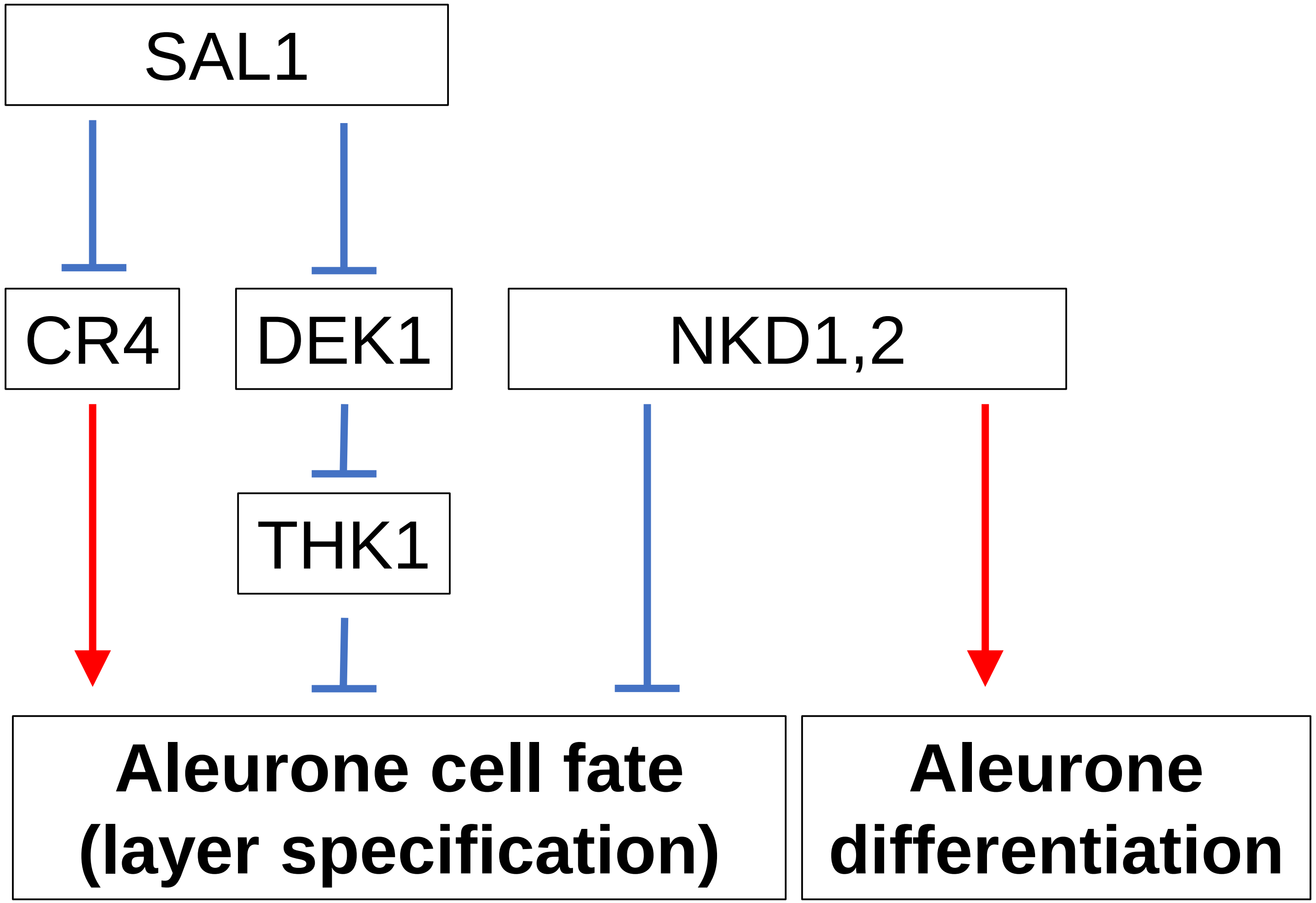

Supplemental Figure 1. Major regulators of maize aleurone development and the relationships among them.

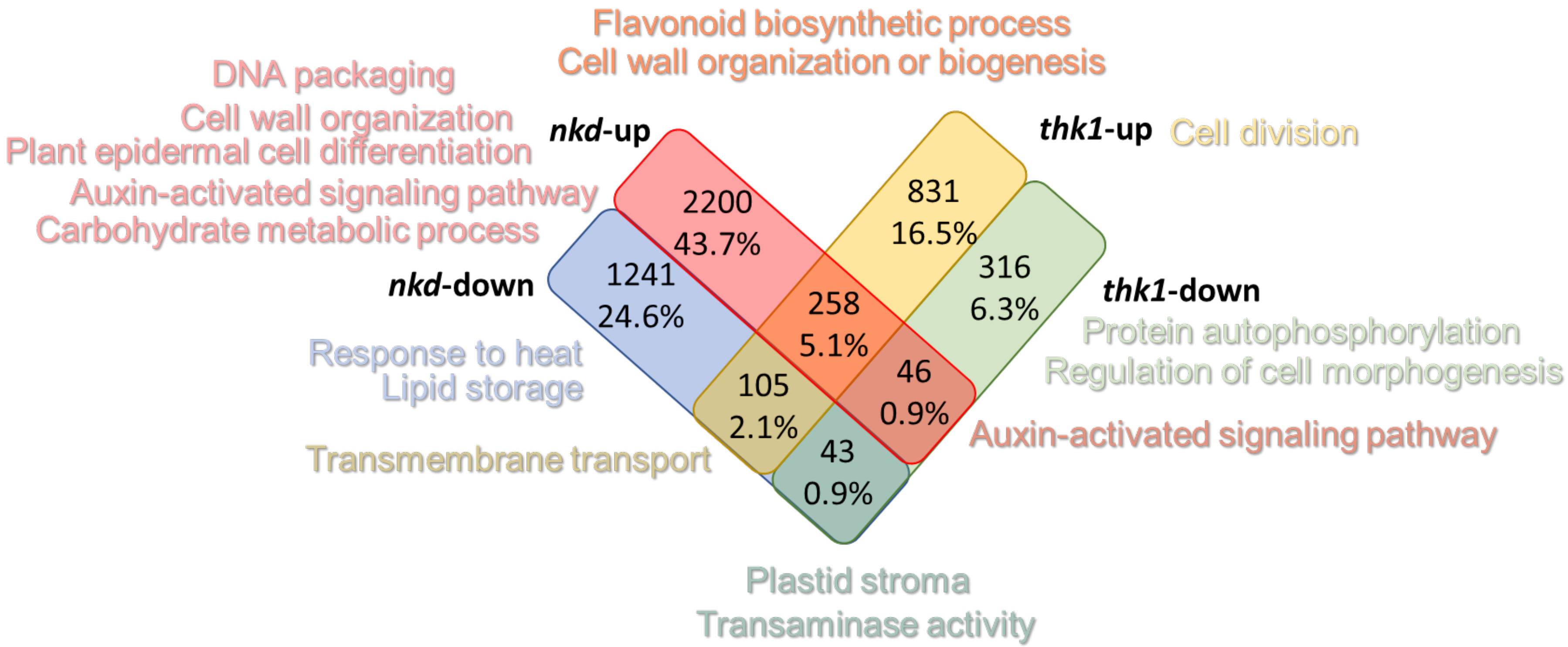

Supplemental Figure 2 Venn diagram showing overlap between DEGs of *nkd1,2* and *thk1* datasets, and GO-terms enriched in each region of overlap.

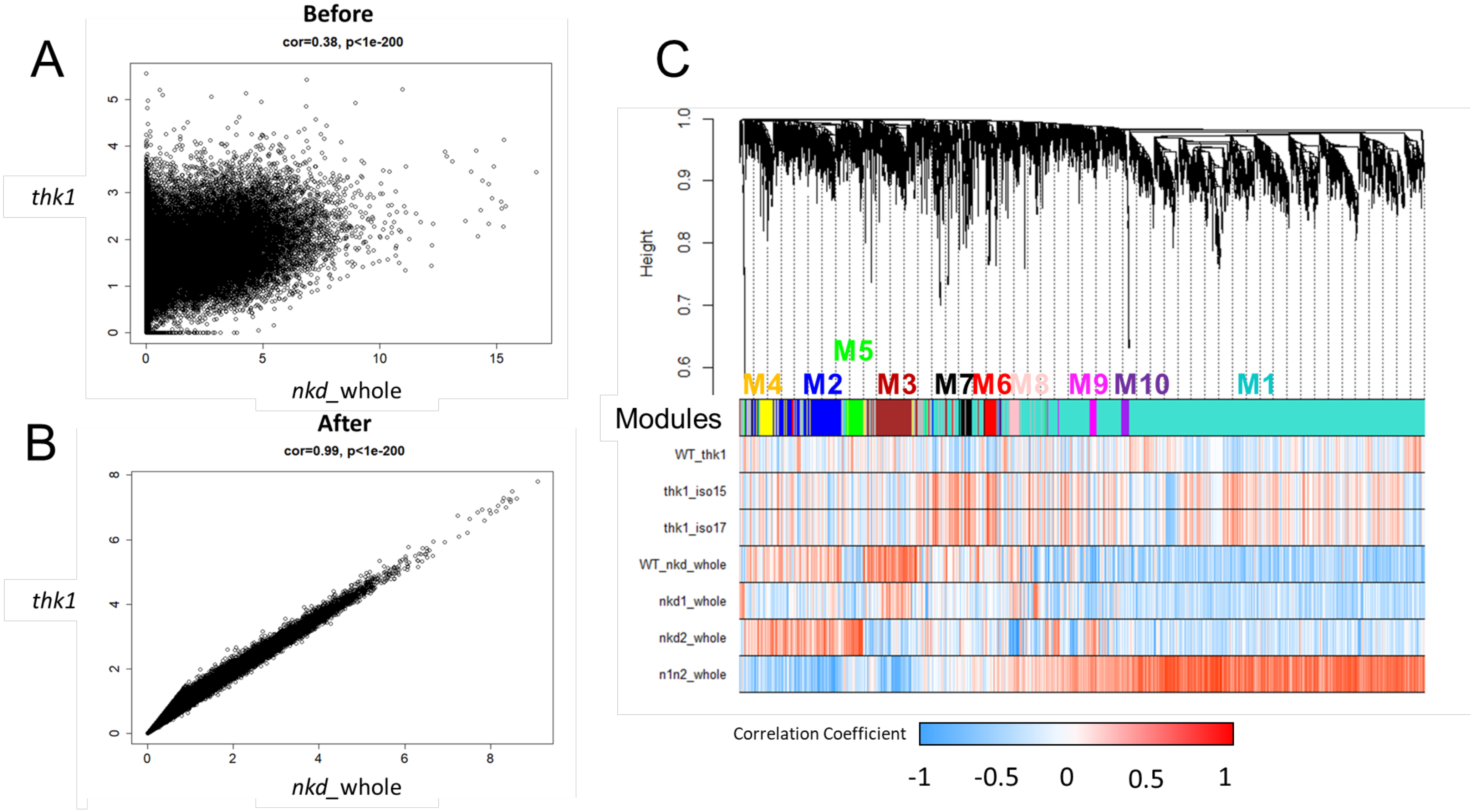

Supplemental Figure 3. Batch effect removal of WGCNA input data and network correlation with genes of each genotype. **(A)** and **(B)** Correlation of *Row_means* between *thk1* WT/mutant dataset (y-axis) and *nkd* WT/mutant dataset (x-axis) before **(A)** and after **(B)** batch effect removal. The *thk1* WT/mutant dataset includes normalized TPM of WT, *thk1*-iso15 and –iso17. The *nkd* WT/mutant dataset includes normalized TPM of WT, *nkd1* or *nkd2* single mutants and *nkd1,2* double mutant. **(C)** Cluster dendrogram of color-coded modules, and correlation of genes with modules.

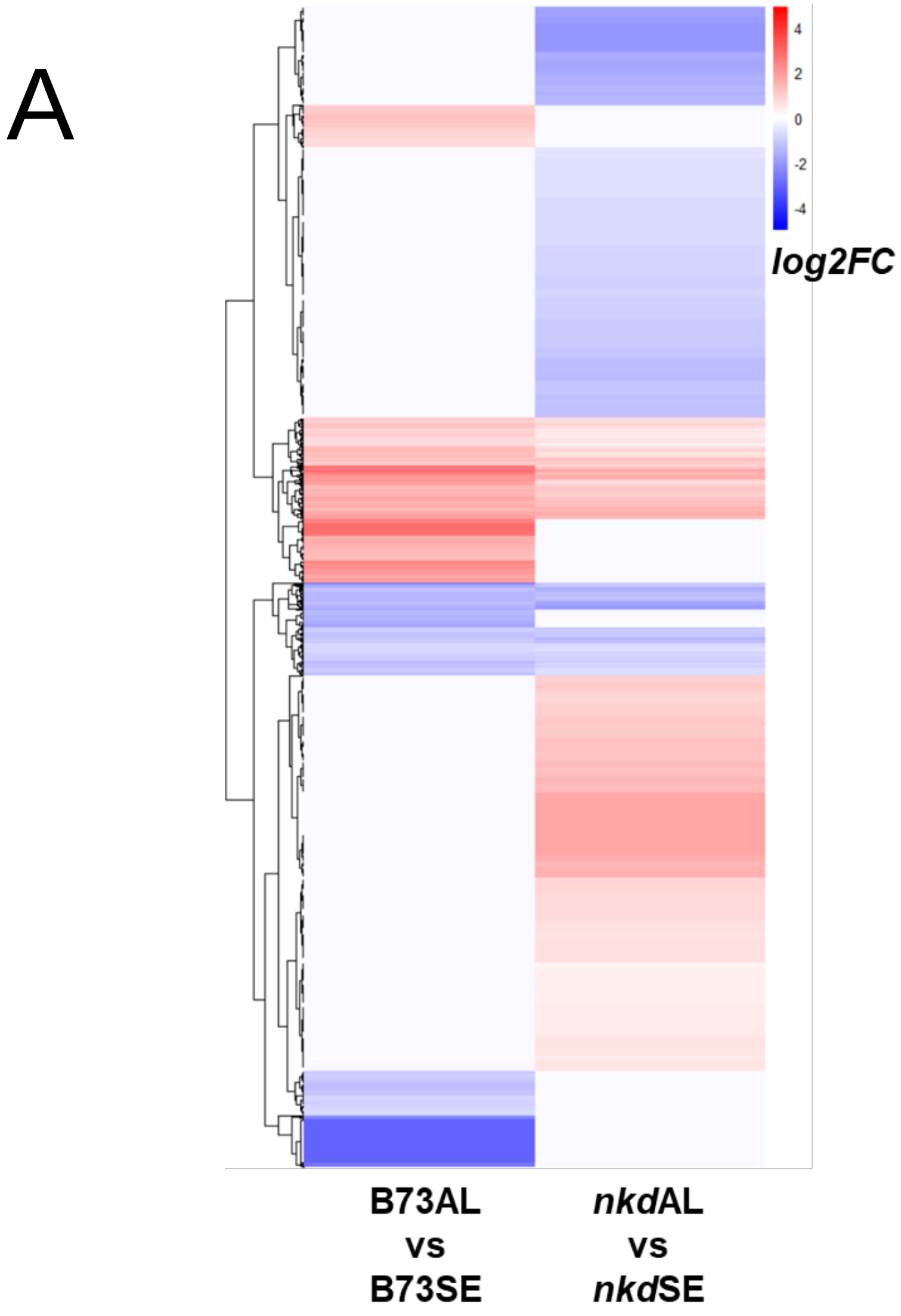

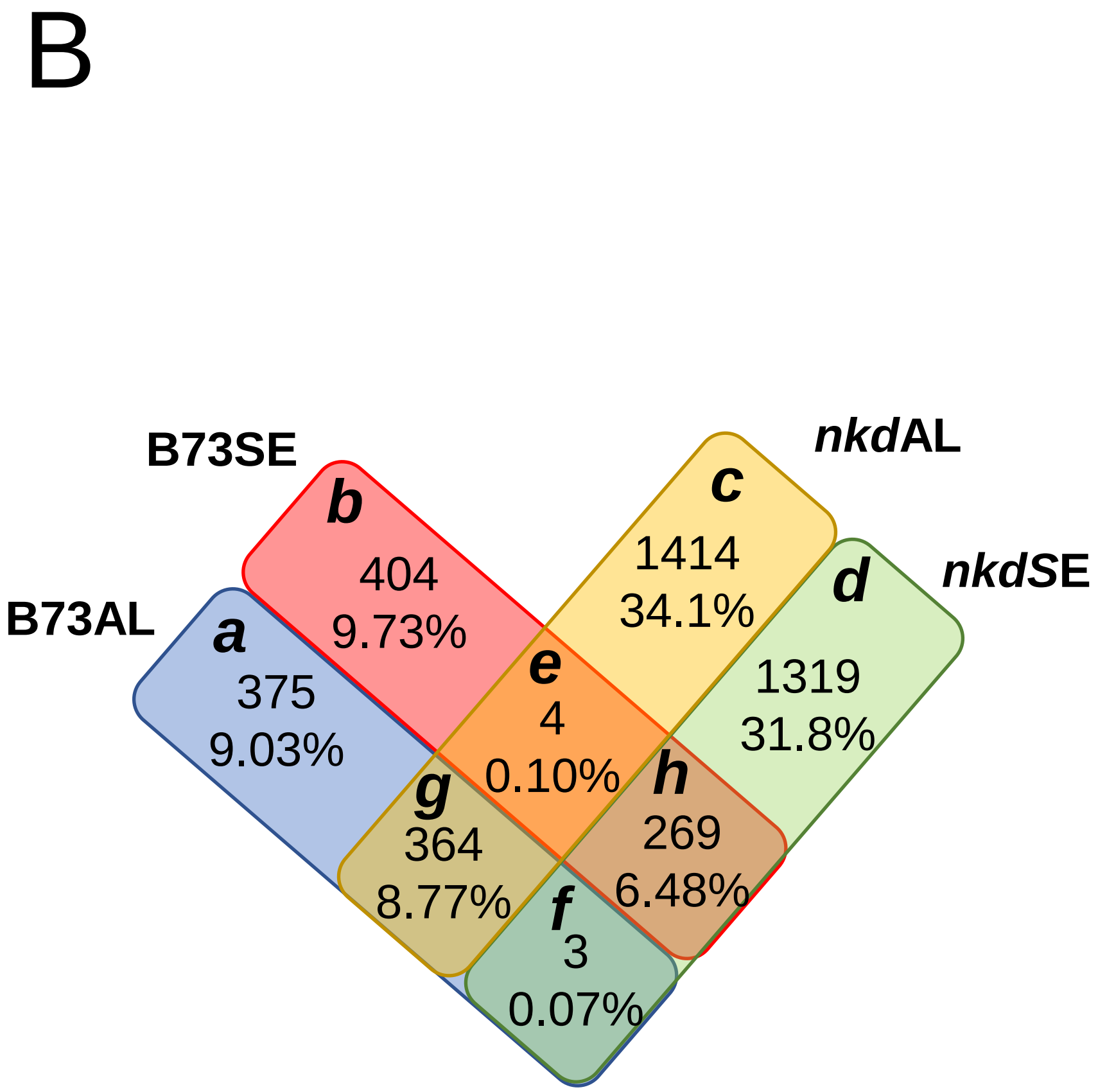

Supplemental Figure 4. Differentially expressed genes between AL and SE by B73 WT and *nkd* backgrounds. **(A.)** Heatmap showing DEGs between AL and SE in B73 WT and *nkd* mutant backgrounds. Red and blue bands represent significantly up- and down-regulated genes (AL- and SE-preferential genes, respectively) within each corresponding genetic background. DEGs are defined as $|log2FC| > 1$ with adjusted p-value < 0.05. **(B.)** Venn diagram showing overlap between DEGs of B73AL vs B73SE and *nkd*AL vs *nkd*SE.

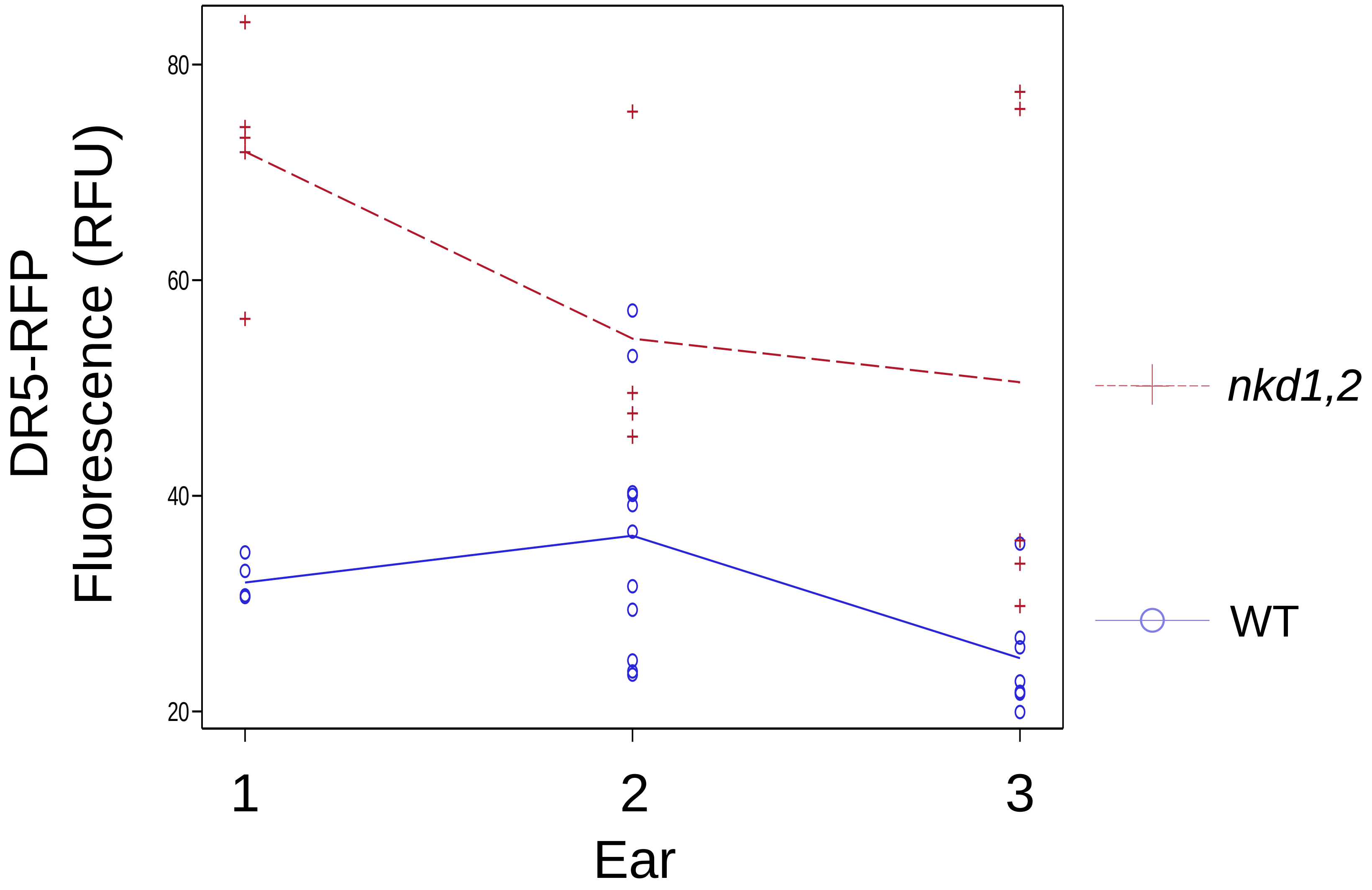

Supplemental Figure 7. Variation in fluorescence levels for the DR5:-RFP transgene in 3 independent ears segregating WT and *nkd1,2* mutant kernels. As shown in the ANOVA interaction plot, there was substantial variation among ears, and among kernels of each phenotypic class within each ear. Mean RFP intensity is consistently higher in *nkd1,2* than WT for each ear.

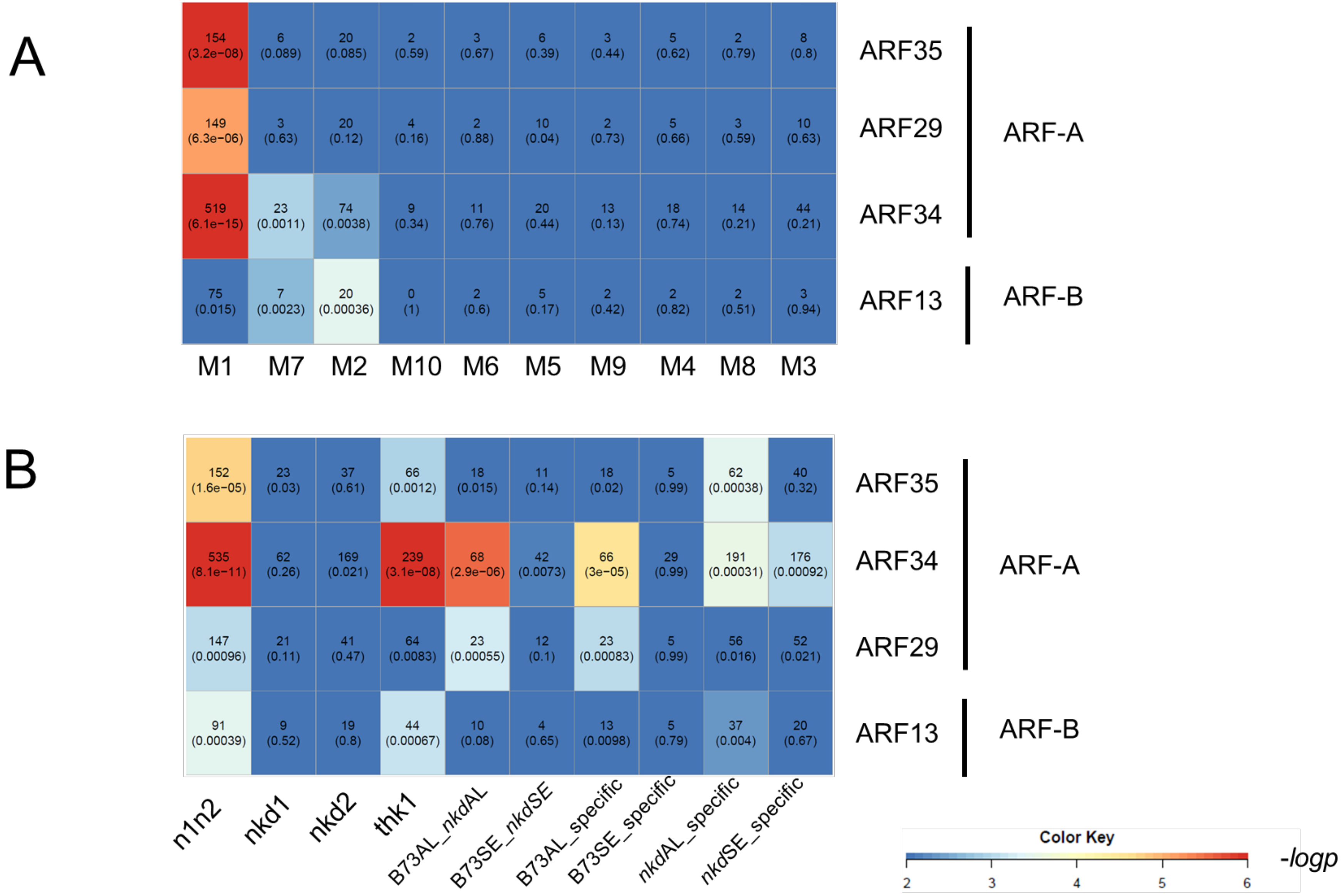

Supplemental Figure 5. Overlap between ARFs versus co-expression modules and DEGs. **(A)** ARFs versus co-expression modules. **(B)** ARFs versus DEGs. Statistical significance of the overlap was tested using a hypergeometric test. The upper number in each cell represents the number of overlapping genes and bottom parenthesized number the corresponding *p*-value.

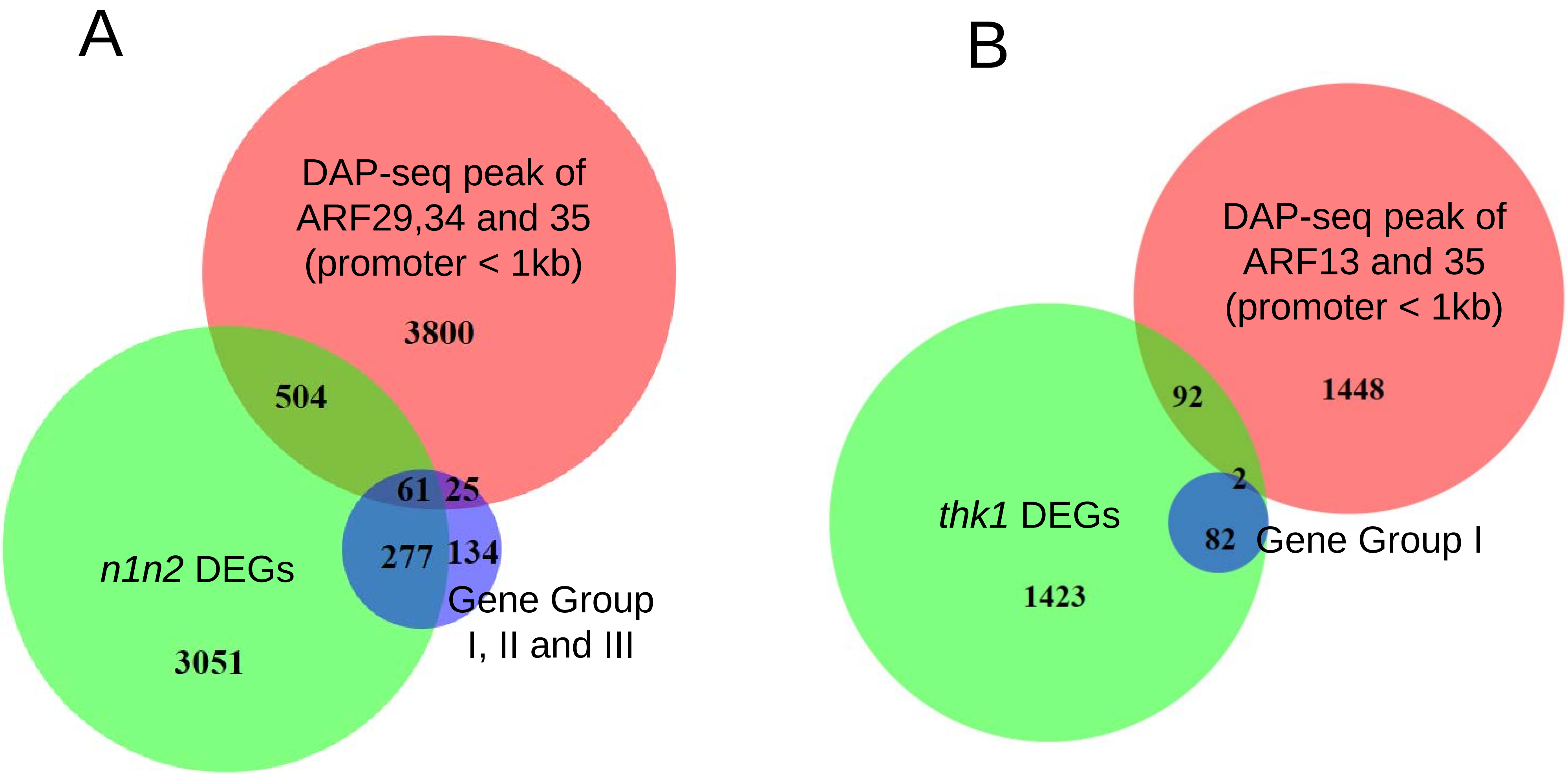

Supplemental Figure 6. Overlap between ARF putative targets, AL preferential genes, and **(A)** DEGs of *nkd1,2* and **(B)** DEGS of *thk1*. Group I, II and III from Fig. 2C.
